# Individual gaze predicts individual scene descriptions

**DOI:** 10.1038/s41598-025-94056-4

**Published:** 2025-03-19

**Authors:** Diana Kollenda, Anna-Sophia Reher, Benjamin de Haas

**Affiliations:** 1 Experimental Psychology, Justus Liebig University, Giessen, Germany; 2 Center for Mind, Brain and Behavior (CMBB), Marburg and Giessen, Germany

**Keywords:** Individual differences, Perceptual consequences, Gaze behaviour, Scene descriptions, Psychology, Human behaviour

## Abstract

Do different people looking at the same scene perceive individual versions of what’s in front of them? If perception is individual, which mechanisms mediate our particular view of the world? Recent findings have shown systematic observer differences in gaze, but it is unclear whether individual fixation biases translate to divergent impressions of the same scene. Here, we find systematic differences in the scene descriptions individual observers provide for identical complex scenes. Crucially, observer differences in fixation patterns predicted pairwise differences in scene descriptions, particularly the use of nouns, even for out-of-sample images. Part of this could be explained by the individual tendency to fixate text and people predicting corresponding description references. Our results strongly suggest that subjective scene perception is shaped by individual gaze.

## Introduction

What does it mean to see with your own eyes? When presented with the same scene, how do our individual impressions diverge from each other? While the subjective quality of perceptual experience is private^1^, neuroimaging work suggests a high degree of observer-similarity in the geometry of ventral stream representations^2^. At the same time, neural responses to identical stimuli can diverge in ways scaling with social distance^3^ and the individual functional anatomy of the visual system predicts low-level psychophysical biases^4,5^. How individual perception varies for complex natural scenes and which mechanisms mediate such differences is less well understood.

When free-viewing complex scenes, human fixations are systematically drawn towards objects, faces, people and text^6–9^. Recent findings show that these general tendencies are modulated by substantial individual variation: Observers show large and robust individual differences in the tendency to fixate faces, people and text as well as other semantic salience dimensions^10–14^. Moreover, twin studies indicate a surprising degree of heritability for individual gaze, suggesting it is shaped by individual biology^13,15^. Do such individual differences in gaze bear consequences for scene understanding, perception and memory?^16^ Previous studies have shown that individual face and head salience are related to skills in face recognition^10,17^ and to prioritising social elements for scene understanding^14^. Nevertheless, it is largely unclear whether individual gaze is systematically related to the individual way we perceive and communicate natural scenes and their content.

At the group level, previous research shows that objects which are critical to scene understanding are fixated (preprint by^18^) and objects which are fixated (longer), in turn are remembered and reported more frequently and in more detail^19–22^. For example, one study demonstrated that the more fixations are made on an object, the better the memory for its position in space^19^. Another study found a positive correlation between the naming of objects in image descriptions and previous fixations on these^22^.

When describing pictures, artwork or our surroundings, verbal communication is a tool to convey our observations to others. Such descriptions can be rich in detail, even for short presentation times^23^. When asking observers to focus on the most relevant aspects and limit their descriptions to one or two sentences, individual descriptions can vary substantially^24^, especially if there are many objects in a scene^21,22^, as is typical for complex natural scenes. A study by Coco & Keller (2012)^25^ combined a target search and sentence production task to investigate the relationship between fixation locations and scene descriptions. Visual attention towards a cued visual target influenced scene descriptions, and descriptions were more similar between participants when the order and location of fixations were also more similar. Importantly, the observed cross-modal similarity for a given scene was greater within an individual than between different individuals. This finding enabled the researchers to predict a participant’s scene description based on their respective gaze pattern. However, it is unclear whether such individual differences in scene descriptions are systematic and reproducible, not only for a particular scene but also across different sets of scenes, as suggested by the findings of trait-like differences in gaze^10,11,26^. We aimed to investigate this gap and hypothesized that stable individual differences in fixation tendencies predict corresponding differences in descriptions^16^.

Here, we find that individual differences in briefly describing 100 natural scenes reflect stable observer differences, which are explained and predicted by individual differences in gaze behaviour towards those scenes. Specifically, we computed pairwise observer similarities, based on the semantic similarity of complete scene descriptions as well as in the use of nouns, verbs or adjectives specifically. Similarly, we quantified pairwise similarities of dwell time distributions across objects in scenes. This abstraction to pairwise observer similarities enabled us to test a relationship between individual gaze and descriptions independent of a priori assumptions regarding the precise mapping of the two modalities onto each other (cf. ref.^27^). Observers with more similar patterns of gaze indeed provided more similar scene descriptions. Crucially, these individual tendencies proved robust enough to decode which observer provided which descriptions, based on individual gaze for a *separate* set of scenes, suggesting trait-like differences in gaze result in trait-like differences in perception. The relationship between gaze and descriptions was particularly evident in the idiosyncratic use of nouns, specifically the tendency to fixate and reference people and text.

## Results

### Observer (dis)similarities in gaze and descriptions are highly consistent across scenes

**Fig. 1:**
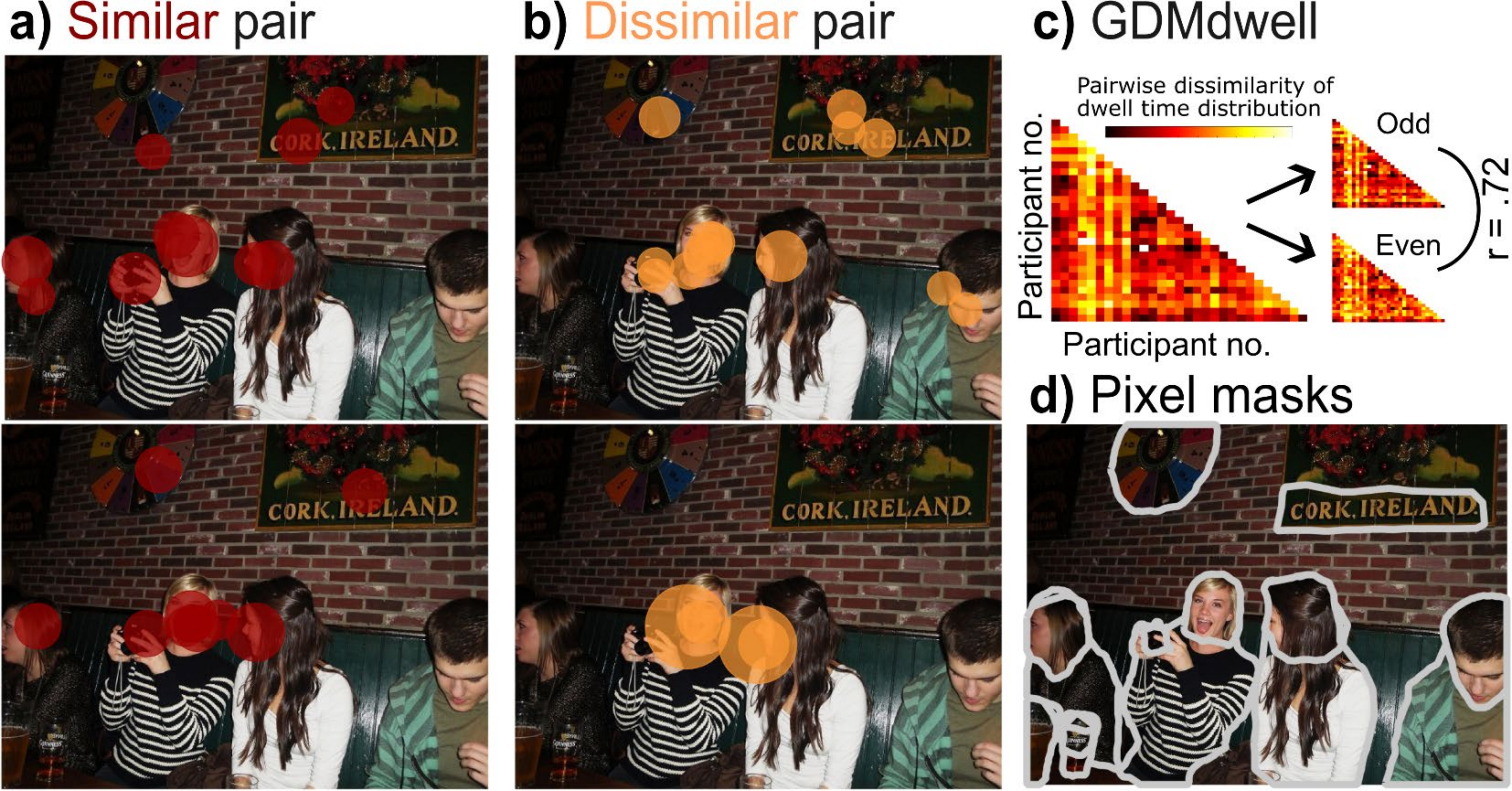
Pairwise dissimilarities in gaze. (**a**) Example fixations from a pair of observers with more similar gaze (participants 1 and 11; dissimilarity score of 0.14) and (**b**) with more dissimilar gaze (participants 9 and 19, dissimilarity score of 0.87). Dissimilarity scores range from 0 to 1 with higher values indicating less similarity. The size of fixation disks scales with fixation duration. (**c**) The gaze dissimilarity matrix (GDM) shows the pairwise observer dissimilarities based on object dwell times. Hotter colours indicate higher dissimilarity scores. The correlation value corresponds to the split-half reliability of the GDM across odd and even trials (p < 0.001). (**d**) The example image was modified from Xu et al.'s (2014) OSIE dataset, which was published under the Massachusetts Institute of Technology license^6^, to display object pixel masks used for calculating object dwell times.

Participants viewed 100 natural scenes for three seconds each and briefly described what they had just seen after each scene. We recorded each observer’s gaze and represented it as a vector of dwell times across all objects and people in the images. We then quantified pairwise observer dissimilarities as the difference between these vectors and entered all pairwise dissimilarities into a Gaze Dissimilarity Matrix (GDM*dwell*). Repeating this separately for odd and even scenes confirmed highly consistent inter-individual gaze dissimilarities across scenes (*r* = 0.72, *p* < 0.001; Fig. 1c; also see Fig. 1a,b for example overlays of pairs with similar and dissimilar gaze tendencies; cf. ref. ^10^). We additionally tested individual differences in the tendency to fixate a given object at all. When calculating pairwise dissimilarities based on binary vectors indicating whether an object was fixated or not (coded as 1 or 0; GDM*fix*), the consistency of inter-individual gaze dissimilarities was reduced, but still significant, *r* = 0.58, *p* < 0.001.

**Fig. 2:**
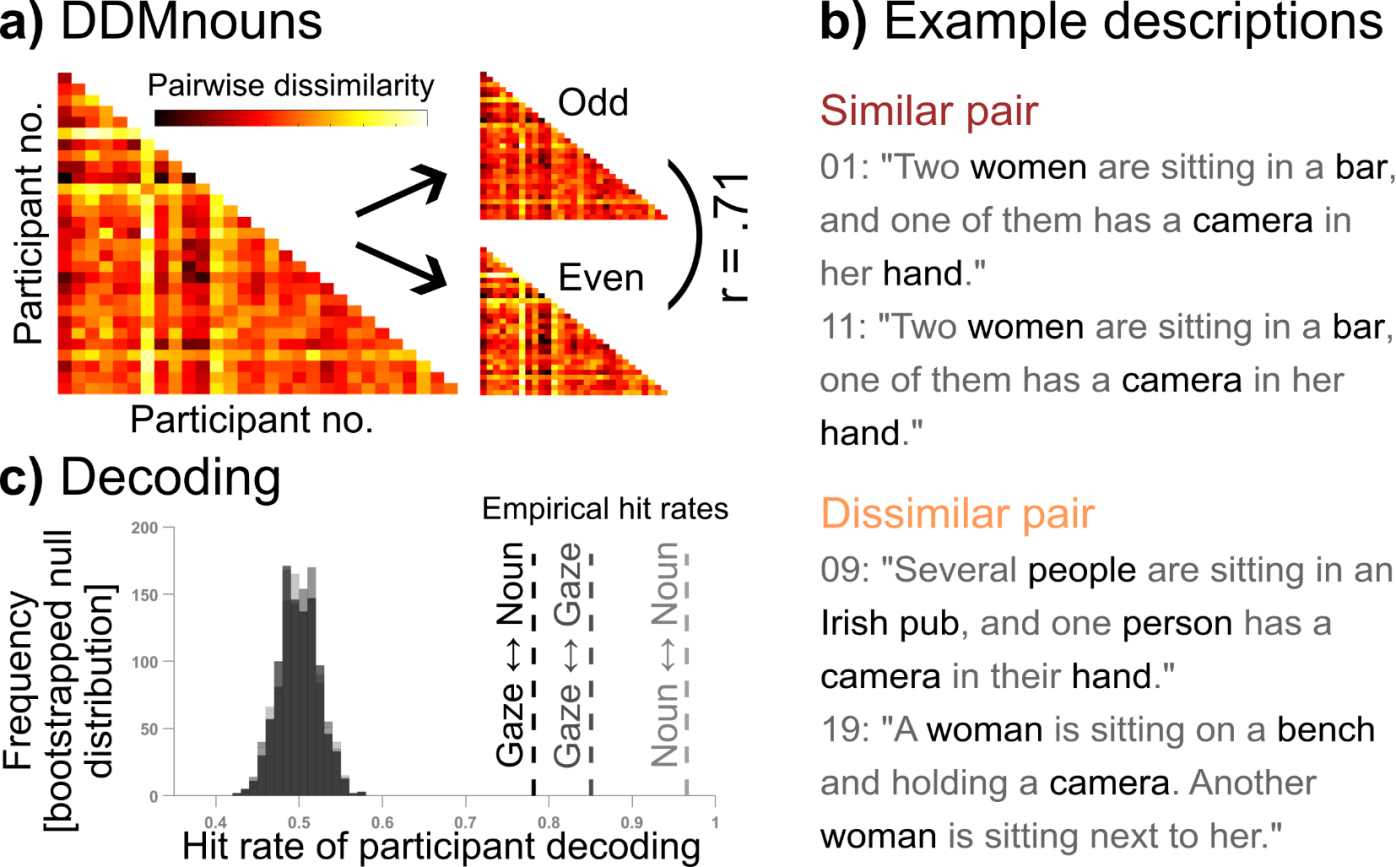
Pairwise dissimilarities in scene descriptions. (**a**) The example description dissimilarity matrix (DDM) shows pairwise observer dissimilarities based on the average semantic similarities of used nouns in descriptions. Higher values are displayed as hotter colours and indicate less semantic similarity. The correlation shows the split-half reliabilities across odd and even scenes, p < 0.001. (**b**) Example descriptions for the scene shown in Fig. 1 from a similar (participants 1 and 11; dissimilarity scores for nouns: 0.39; verbs: 0.34; adjectives: 0.2) and dissimilar pair of observers (participants 9 and 19; dissimilarity scores for nouns: 0.72; verbs: 0.48; adjectives: 0.35). The values range from 0 to 1 with higher values indicating higher dissimilarity. All sentences were translated to English and the original German versions can be found in Supplementary Information B. (**c**) The vertical lines represent the empirical hit rates for decoding individual observers based on out-of-sample training data from different sets of scenes and are categorized by decoding of gaze <—> gaze (dark grey), noun <—> noun (light grey), and gaze <—> noun (black). Each hit rate is compared against its respective bootstrapped null distribution.

Similarly, we quantified pairwise *description* differences as one minus the semantic similarity of scene descriptions. We did this separately for complete scene descriptions as well as for the similarity of used nouns, verbs or adjectives. To derive a quantitative estimate of semantic similarity, we determined the cosine similarity of description embeddings using SentenceBERT^28^ for complete descriptions and the genSim library^29^ and pretrained fastText model^30,31^ for pairs of words (see methods for details). We then averaged the resulting difference values across scenes for each pair of observers to compute Description Dissimilarity Matrices (DDMs). Split-half correlations confirmed high internal consistencies for the DDMs for complete descriptions, *r* = 0.79, *p* < 0.001, nouns, *r* = 0.71, *p* < 0.001; verbs, *r* = 0.95, *p* < 0.001; and adjectives, *r* = 0.71, *p* < 0.001 (see Fig. 2a-b for an example). Thus, pairwise dissimilarities in descriptions reflect systematic observer differences, which generalize across scenes. Notably, we found highly consistent differences in the use of all word types, even though their average frequency per description varied considerably (nouns: *M* = 3.88, *SD* = 1.29; verbs: *M* = 1.91, *SD* = 0.77; adjectives: *M* = 0.53, *SD* = 0.65).

Taken together, our findings confirm that pairwise similarities in both, gaze and scene descriptions are stable across separate sets of scenes.

### Gaze similarity explains description similarity

In order to test the main hypothesis that individual gaze predicts individual descriptions, we correlated the gaze dissimilarity matrix with the description dissimilarity matrices for complete descriptions and each word type. In line with our hypothesis, we observed positive correlations between the dissimilarities for dwell time distributions (GDM*dwell*) and complete scene description (DDM*complete*), *r* = 0.33, *p* < 0.001, as well as nouns (DDM*noun*), *r* = 0.42, *p* < 0.001, but not verbs, *r* = -0.07, *p* = 0.128 and adjectives,* r* = -0.06, *p* = 0.210. Although individual differences in which objects were fixated at all (GDM*fix*) are less consistent (see above), GDM*fix* correlated positively with dissimilarities in noun use DDM*noun*, *r* = 0.38, *p* < 0.001 and to a lesser extent with dissimilarities in complete descriptions DDM*complete*,* r* = 0.21, *p* < 0.001. All remaining analyses focus on dissimilarities in noun use, but we will return to the use of verbs and adjectives in the discussion section.

To further test the relationship between individual gaze and scene descriptions, we tested whether observer identity can be decoded via dissimilarity patterns across independent sets of scenes. Specifically, we aimed to decode which of two observers provided a given set of fixations or descriptions, based on their dissimilarity to other observers. This tests two assumptions: 1) Every observer has a fairly unique pattern of gaze and description dissimilarity compared to other observers; 2) This unique pattern of dissimilarity generalizes across independent sets of scenes. When splitting the gaze data into those from odd and even scenes, we were able to decode which of two observers provided a given dwell time distribution based on the nearest neighbor correlation of dissimilarity patterns with those observed for the other half of the data (GDM*dwell*; gaze <—> gaze decoding hit rate 85%, *p* < 0.001). The same applied to decoding observer identity based on the dissimilarity pattern of descriptions (DDM*noun*; description <—> description decoding hit rate 97%, *p* < 0.001). Crucially, inter-observer differences did not just generalize across scenes, but also from gaze to descriptions. Decoding which of two observers provided a given set of scene descriptions (or gaze patterns) also worked based on a nearest neighbor correlation between their scene description dissimilarities with their dwell-time dissimilarities for another set of scenes (GDM*dwell* and DDM*noun*; gaze <—> description decoding hit rate of 78%, *p* < 0.001; see Fig. 2c). All p-values were derived from a statistical comparison of the respective empirical hit rate with their bootstrapped null distribution in a permutation analysis (see Methods for details).

Taken together, individual differences in gaze predict individual differences in descriptions, which generalize to hold-out scenes.

### The tendency to fixate text and people explains the tendency to reference them in descriptions

**Fig. 3:**
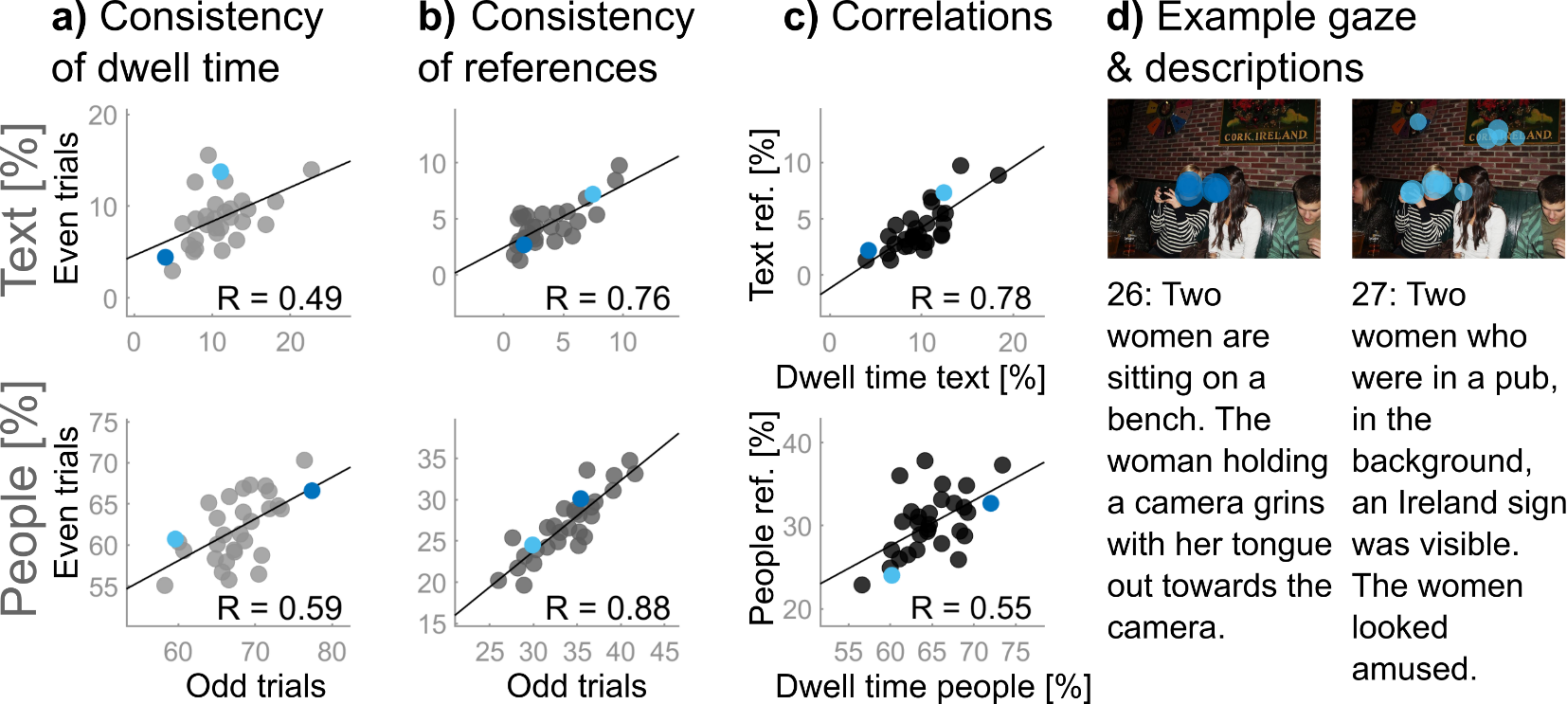
Fixations predict references to text and people. Correlations show (**a**) consistency of individual dwell time allocations across odd and even trials for text elements, p = 0.006, and people, p < 0.001, as well as (**b**) consistency of individual references in descriptions across odd even trials for text, p < 0.001 and people, p < 0.001. Correlations in (**c**) show consistency of individual differences across dwell time allocations and references to text, p < 0.001 and people, p = 0.002. Coloured dots in each scatter highlight data from the two example participants (26 and 27) whose fixation pattern and descriptions are shown in (**d**). The original German sentences can be found in Supplementary Information B.

Previous studies found consistent individual differences in the tendency to fixate text and people^10–12,14^, which replicated in our experiment (split-half reliabilities of *r* = 0.49, *p* = 0.006, and *r* = 0.59, *p* < 0.001, for text and people, respectively). Some observers spent more than four times (factor of 4.66) as much of their dwell time on text than others and the proportion of dwell time spent on people varied up to a factor of 1.3 (cf. Figure 3a). Furthermore, we found a negative correlation between the individual proportion of dwell time on text and that on people, *r* = -0.74, *p* < 0.001, again in line with earlier results.^10^.

Interestingly, individual differences in the proportion of text and people references in *descriptions* were also highly consistent, (*r* = 0.76, *p* < 0.001 and *r* = 0.88, *p* < 0.001, respectively), with some observers referencing text 7.36 as often as others and some observers referencing people 1.65 as often as others (cf. Figure 3b). Again, individual differences in the proportions of text and people references were negatively correlated with each other, *r* = -0.58, *p* < 0.001. Crucially, the individual proportion of dwell time on text was highly correlated with the individual proportion of text references in descriptions, *r* = 0.78, *p* < 0.001, and the same was true for the individual proportion of dwell time on people and the proportion of people references in descriptions, *r* = 0.55, *p* = 0.002 (cf. Figure 3c). That is, a large fraction of the variance in referencing text and people in scene descriptions could be explained by corresponding fixation biases (see Fig. 3d for a gaze and description example).

These results suggest that individual tendencies to fixate and reference text and people are an important part of the more general relationship between individual differences in dwell time distributions (GDM*dwell*) and descriptions (DDM*noun*). In an additional analysis we tested whether this relationship can be *reduced* to individual fixation biases towards text and people or captures additional covariance. To juxtapose these possibilities, we computed additional gaze dissimilarity matrices for the pairwise differences in the proportion of dwell time on text (GDM*text*) and people (GDM*people*) for which we obtained internal consistencies of respectively *r* = 0.31, *p* < 0.001 and *r* = 0.64, *p* < 0.001. We then fitted a multiple linear regression model with the general GDM*dwell*, GDM*text* and GDM*people* as predictors of the DDM*noun*, yielding an adjusted *R*^2^ of 0.18, *F*(3, 431) = 32.2, *p* < 0.001. Dropping the general GDM from the model resulted in a significant decrease of model performance (adjusted *R*^2^ of 0.15, *F*(2, 432) = 39.1, *p* < 0.001, ΔAIC = 13.55), revealing a unique contribution of general dwell-time similarities to description similarities beyond individual tendencies to fixate people and text. This suggests that our general and targeted analyses are not redundant and the relationship between individual gaze and descriptions contains further dimensions yet to be identified.

## Discussion

We investigated systematic individual differences in gaze and found that they translated to individually divergent descriptions of identical scenes.

We observed positive correlations between pairwise observer dissimilarities in scene descriptions and fixation patterns. This relationship was strongest for the semantic dissimilarity of nouns and not significant for verbs and adjectives. Nouns most directly match the objects across which we quantified individual fixation distributions. To test whether the use of object masks for GDM*dwell* biased results towards DDM*nouns*, we conducted an additional control analysis using pixel-based fixation density maps instead (see Supplementary Information C). Reassuringly, this analysis yielded the same pattern of results, reinforcing our conclusion that inter-individual gaze similarity aligns with the usage of nouns, but not verbs or adjectives. Moreover, recent results suggest that scene representations in higher visual cortices are well captured by the nouns used in corresponding descriptions^32^. Semantic similarity of complete scene descriptions (containing nouns, verbs, adjectives and function words) also correlated positively with fixation patterns, but less strongly than that of nouns. This is further support of the prominent role of nouns in explaining fixation patterns.

Although verbs and adjectives are important for scene understanding, e.g., by disambiguating correspondences^33^ and describing relationships, details and actions, it is less clear which pixels in an image or fixations in a gaze pattern would correspond to them. This may be the reason why previous research comparing gaze and descriptions focused on nouns^21,22^. Interestingly, however, we also observed consistent individual differences in the verbs used in scene descriptions. Future work may quantify action-related differences in gaze more directly. For instance, recent work showed that deep saliency models appear to use semantic action features (e.g. having a meeting, snowboarding, and jumping) to predict visual attention^34^. It may be possible to estimate the weight of such features on an individual level and relate them to scene descriptions. Additionally, verbs could be grouped into functional categories^35^ which semantically link objects, people, or both. Individual differences in the use of such verb clusters may be related to corresponding differences in scan path sequences^16^.

Probing more specific biases, reliable individual differences in the tendency to look at people and text^10–12,14^ explained corresponding references in scene descriptions. This extends the previous finding that objects which are more frequently fixated are also more likely to be referenced^19–22^ to the level of trait-like individual differences. The *habitual individual* way we look at scenes is predictive of the individual way we communicate their content. Importantly, this variation generalised across independent sets of scenes. How an individual looks at one set of scenes is predictive of how it will describe another. This strongly suggests that individual gaze is linked to trait-like differences in subjective scene perception. The interindividual variance in the proportion of text references and dwell time was very large (ratio of extremes close to factor seven and five, respectively) and that for the proportion of people references and dwell time was smaller (ratio of extremes 1.65 and 1.3, respectively). This resonates with previous findings showing that people and faces have a high probability to be fixated and included in scene descriptions overall^22^ (see also ref.^36^), which limits the scale of variation due to ceiling effects. In contrast, reading is an acquired skill (e.g.,^37^) and text salience may be modulated by education, profession and experience throughout life. Because the group of participants we studied has similar educational backgrounds, the variation in text salience and references may be even larger in more diverse samples. Generally, our study sample was relatively homogeneous, consisting mainly of female German university students. Future studies could use the methods introduced here to study variance across more diverse groups including different educational and cultural backgrounds, children, experts and individuals with clinical conditions, who may perceive and describe scenes differently. Previous studies indicate altered viewing patterns in autism spectrum and affective disorders, such as reduced visual exploration and social salience^38,39^. However, the connection between these gaze characteristics and scene perception remains to be tested.

A potential limitation of our findings is that it is unclear how far they generalise to other tasks than describing scenes. Interestingly, our paradigm replicated the most important and reliable individual fixation tendencies previously found in free-viewing tasks (i.e. anticorrelated tendencies to fixate text and faces). This speaks against a narrow task-dependence of these individual tendencies. Additionally, the reliability of these gaze tendencies across time and independent sets of scenes^10,11^ suggests they reflect ecological meaningful observer traits. Furthermore, a recent preprint by Murlidaran and Eckstein (2024) found similar patterns of fixations when instructing participants to freely view or describe a scene (but see ref.^40^) and suggests the extraction of global meaning as the default objective of scene viewing. This supports the notion that our description task is suitable to study ecologically valid individual differences in perception. Nevertheless, further research is needed to investigate the robustness of the observed individual differences across tasks and dynamic or real-world stimuli.

Furthermore, there may be aspects of gaze that correlate with nouns, verbs, and adjectives when examined more closely, though we have yet to identify them. A post-hoc exploratory analysis (see Supplementary Information D) suggested that observer dissimilarities in the tendency to fixate “touched” objects was positively correlated not only with noun dissimilarity patterns, but also with description differences in verbs and adjectives. These findings provide a promising starting point for future research, which could further explore these relationships using larger image sets. Finally, while our results provide evidence for systematic individual differences in scene descriptions and for individual gaze as a mediating mechanism, they cannot reveal why people attend to different things in the first place. Twin studies found high heritability for individual differences in gaze^13,15^, and experiences like learning to read appear to shape scene viewing as well^41^. Genetic differences may imply structural and functional differences in the visual system^42^, which in turn may bias individual visual diets and ultimately lead to the individual differences in scene perception suggested by our results. Future studies can probe individual gaze and scene descriptions (or drawings^43^) in large and age-diverse cohorts to decipher their developmental relationship.

Our findings open a window into individual perception which is potentially relevant beyond biology and vision science. For instance, individual gaze may shape the appreciation of visual stimuli and memorability of artworks (cf. ref.^44^) or contribute to the strikingly individual appraisal of motion pictures^45^. Systematic covariance, such as the anticorrelation between text and people salience point to the possibility of a low-dimensional space of individual perception (cf. ref.^10^), which could inform the design of visual communication from advertisement to education and road safety.

In sum, we found consistent differences in how individuals describe identical natural scenes, which can be explained by corresponding differences in gaze. These results strongly suggest that the individual way we see the world is shaped by the individual way we look at it.

## Methods

### Participants

We included 30 participants (average age: *M* = 22.77; *SD* = 2.87; 4 male, 26 female), recruited from the participants mailing list at Justus-Liebig University Giessen, Germany, with normal or corrected-to-normal vision, German language proficiency at native level and no acute mental health problems. Participants were compensated with course credit or 20 euros.

The study was approved by the local ethics committee of the Justus-Liebig-University Giessen (LEK-FB06) and was conducted in accordance with the relevant guidelines and regulations. All participants provided written informed consent.

### Apparatus

Data were collected using Psychtoolbox^46^ and MATLAB (R2020a, MathWorks, Natick, MA) on a BenQ XL2430T computer monitor (BenQ Corporation, Taipei, Taiwan). The dimensions of the display, which was placed at a distance of 55 cm, were 526 × 296 mm with a 3840 × 2160 pixel resolution. Stimuli were presented at a size of 2400 × 1800 pixels and 34.3 × 25.7 degrees viewing angle. Movements of the right eye were recorded at a frequency of 1 kHz using a towermount Eyelink 1000 eye tracker (SR Research, Ottawa, Canada).

### Stimuli and procedure

Participants were presented with a total of 100 images from the OSIE image set^6^ in fixed order. These images depicted everyday natural indoor and outdoor scenes and came with a total of 845 pixel masks (published by Xu et al., 2014) for various objects ranging from inanimate to animate objects (see Fig. 1d for an example). For our analysis focusing on specific object categories, we considered 84 pixel masks for “text” elements and 237 pixel masks for “people” (the latter were published by Broda & de Haas (2022)[26]). The selection of the 100 images for our experiment was composed of 40 everyday scenes that were found to be particularly suitable for analysing individual gaze behaviour in the study by Linka & de Haas (2020)^11^ (cf. OSIE40) as well as 60 additional scenes taken from the OSIE200^11^, which were selected with a preference for dynamic content (all stimuli can be found at [https://osf.io/83mjc]).

After providing written informed consent, participants were given written instructions, familiarized with the procedure and provided the opportunity to ask questions. Instructions (Supplementary Information A) loosely followed Chen et al. (2015)^24^ and asked participants to explore and describe each image in turn (cf. ref.^10^). Participants initiated a trial by simultaneously fixating a fixation point at the centre of the screen and pressing the space bar. This triggered the presentation of a scene image which lasted three seconds, followed by a written instruction to describe the image verbally. Participants’ descriptions were documented by the experimenter in writing into an Excel spreadsheet using a laptop. The instructions were presented until the participant pressed the space bar to indicate that they completed their description and to move on to the next trial.

Participants could take short breaks after every 10 trials, followed by a nine-point calibration of the right eye, including validation with a maximum error of 1.2 and a maximum average error of 0.6 (observed validation error: *M* = 0.35 dva, *SD* = 0.06 dva). On average, the experiment lasted 90 to 120 min in total.

### Measured variables and preprocessing

#### Similarity of gaze behaviour

Following de Haas et al. (2019) and manufacturer recommendations, we excluded fixations with an onset latency or duration < 100 ms. We then quantified the cumulative fixation duration (i.e. dwell time) for each observer and object for which we have pixel masks in a given scene (see Fig. 1d for an example scene in which we visually outlined respective pixel masks obtained from ref. ^6^). Moreover, for each observer, we quantified the percentage of overall dwell time across all images which fell on text elements and people, respectively. To account for foveal extent and eye tracking accuracy, a fixation was assigned to each object for which the distance between the fixation coordinate and pixel mask was within a tolerance margin of ∼0.5 degrees visual angle. Note, also multiple assignments were possible for neighbouring or overlapping pixel masks such that a given fixation duration could be considered for more than one object. Dwell times for single objects were then expressed as the proportion of cumulative dwell time across all fixations in the respective scene; dwell times for object classes (people and text elements) were expressed as the proportion of cumulative dwell time across all fixations in all scenes (cf. ref.^10,11^).

Furthermore, the proportion of cumulative dwell times for all 845 objects (pixel masks by ref.^6^) across all 100 images were entered into a vector and compared between each possible pair of observers, using the Euclidian distance as a summary measure of pairwise differences. That is, we computed the squared difference between dwell times of observer A and B for each object and then summed these differences across objects. This measure of difference was computed for all possible pairs of observers and entered into a Gaze Dissimilarity Matrix (GDM). This procedure was repeated, considering object pixel masks with a text (GDM*text*) or people label (GDM*people*) only. Additionally, we calculated pairwise dissimilarities based on the Euclidian distance between binary vectors indicating whether an object was fixated (coded as 1) or not (coded as 0; GDM*fix*). Finally, dissimilarities were scaled to a range from 0 to 1. Note that the GDMs reported here were calculated based on gaze behaviour across all 100 images. However, to assess the internal consistency of the resulting matrices, we later conducted a split-half analysis for which we computed independent GDMs for the 50 odd and 50 even trials. The split-half data (odd and even trials) were also used in the decoding analysis (see the Analysis section for details).

#### Similarity of scene descriptions

To analyse the similarity of scene descriptions, we first quantified the semantic similarity between participants’ complete scene descriptions using the pre-trained paraphrase-MiniLM-L12-v2 model from the Sentence Transformers library (SentenceBERT; ref.^28^) in Python (v. 3.8.17). This model is well-suited for generating embeddings of sentences and computing their semantic similarity based on the cosine similarity between them (ranging from -1 to 1, with higher values indicating higher semantic similarity). For each image, a 30 × 30 similarity matrix was initialized to store the pairwise similarity scores between participants’ descriptions.

Second, we pooled all descriptions for a given image across participants and automatically identified all unique nouns, verbs and adjectives using the part-of-speech tagging by the spaCy library (v. 3.6.0) and the associated German language model in Python (v. 3.8.17). The assignment was subsequently checked manually, and isolated assignment errors were corrected. Moreover, we estimated the semantic similarity of all possible word pairs of the respective scene descriptions by using the genSim library^29^ and the pretrained fastText model^30^, which offers also German language applications^31^ and calculates the similarity values using cosine similarity (ranging from -1 to 1; the higher the value, the higher is the contextual similarity of the words). Finally, we identified the individual words that a subject used to describe a scene and compared the semantic similarity between the words used by this subject and their respective comparison partner. If an identical word was used in a comparison pair, the word was assigned a semantic similarity of 1. If this was not the case, we searched for the semantic ‘nearest neighbour’ among the words used by the comparison partner according to the fastText model, and then assigned the corresponding similarity value to the respective word. We then calculated the mean semantic similarity value across all ‘closest matches’ for a given pair of descriptions. Subsequently, we computed the average similarity of scene descriptions across all scenes for a given pair of observers. Finally, these values were scaled to a range from 0 to 1 across all observers and subtracted from 1 so that higher values were associated with more dissimilar pairs. The resulting Description Dissimilarity Matrices (DDMs) for nouns (containing all data or data from the odd and even splits) are displayed in Fig. 2a. Additionally, we grouped all the nouns referring to text elements and people in a separate text file and quantified the proportion of nouns referring to the respective category for each observer.

## Analysis

We performed all statistical analyses and created all figures using MATLAB (R2020a, MathWorks, Natick, MA).

Computationally, both, gaze patterns and scene descriptions can be thought of as vectors in high-dimensional spaces, defined by the distribution of dwell time across objects in an image, or the semantic similarity of complete descriptions and words from a library of scene descriptions. Our rationale was to map gaze and description data onto each other via the transformation of both variables into the same abstract space of pairwise observer similarities (see above). This is akin to representational similarity analysis in neuroscience, which has been used successfully to compare representational geometries across modalities^27^. It has the additional advantage of requiring no a priori knowledge of the most relevant dimensions of individual variance. While previous research has revealed some specific individual gaze biases^10^, these may not be exhaustive and individual biases for scene descriptions were unknown a priori.

Once the data were transformed to Gaze or Description Dissimilarity Matrices (GDM or DDM), we assessed the internal consistencies of all corresponding matrices (GDM*dwell*, GDM*text*, GDM*people*, GDM*fix*, DDM*complete*, DDM*noun*, DDM*verb*, DDM*adjective*). To obtain a measure of internal consistency, we first split the data into 50 odd and even trials and computed all variables of interest separately for these two halves. The resulting estimates were then compared using split-half correlations. Furthermore, we calculated the inter-subject range (maximum/minimum ratios) of proportions of dwell times and references to text and people (cf. ref.^11^ and ref.^10^).

To test the relationship between individual gaze behaviour and scene descriptions and differentiate between contributions from the general GDM and the a priori dimensions of text and people, we performed a multiple regression with GDM*dwell*, GDM*text* and GDM*people* as predictors and DDM*noun* as target variable. We then dropped each predictor in turn and tested the effect on the adjusted proportion of variance explained by the model (adjusted R^2^) and Akaike Information Criterion (AIC) to assess its unique contribution. A ΔAIC > 4 between the full and reduced model was considered indicative of a significant difference.

To decode an observer’s identity based on their dissimilarity pattern for a set of images, we derived pairwise difference in gaze (GDM*dwell*) and descriptions (DDM*noun*) independently for two halves of the data (odd and even trials). We then extracted the dissimilarity patterns of two observers to all other observers for both halves of the data. Crucially, we provided a decoding algorithm with the corresponding observer labels only for the dissimilarity vectors for one half of the data and tried to determine the observer labels for the corresponding dissimilarity vectors from the other half of the data using nearest neighbour correlations. This was done both, within and between modalities (gaze <—> gaze, noun <—> noun, and gaze <—> noun dissimilarity vector correlations). Empirical hit rates corresponded to the proportion of instances in which the algorithm correctly identified observer identities applying this method across all possible pairs of observers. Finally, we performed 1000 bootstrap iterations to generate a null distribution for the respective hit rates by shuffling observer identities within each split. This allowed us to assess the statistical significance of our empirical hit rates against a baseline distribution obtained under the null hypothesis (see Fig. 2c).

When multiple comparisons were performed, the significance level was adjusted according to Bonferroni by dividing it by the number of comparisons. P-values remain unaffected by this procedure and a correction is only applied in the interpretation of these values with regard to the adjusted significance level. All data and code is publicly available at [https://osf.io/83mjc].

## Supplementary Information

Supplementary Information.

## Data Availability

The datasets generated and analysed in the current study are available for download from the first author’s (Diana Kollenda) OSF profile [https://osf.io/83mjc] along with the analysis scripts and all stimuli.
