# Supplementary material for: Individual gaze predicts individual scene descriptions
Source: Sci Rep. 2025 Mar 19;15:9443. doi: 10.1038/s41598-025-94056-4 (PMC11923161; doi:10.1038/s41598-025-94056-4)
Supplement: Supplementary file 1 — Supplementary Information. [file 41598_2025_94056_MOESM1_ESM.pdf]

Supplementary Information for the manuscript entitled "Individual gaze predicts individual scene descriptions"

Diana Kollenda*, Anna-Sophia Reher & Benjamin de Haas

Supplementary Information A

German original instructions

Wichtig ist, dass Du deinen Kopf in der Kinnstuetze moeglichst nicht bewegst.

In jedem Durchgang siehst Du zuerst ein Fixationskreuz. Sobald Du darauf schaust und die Leertaste drueckst, wirst Du fuer 3 Sekunden ein Bild sehen, das Du frei betrachten kannst.

Im Anschluss bitten wir Dich, dieses Bild muendlich zu beschreiben und ich werde Deine Beschreibung aufschreiben. Es gibt keine richtigen oder falschen Antworten.

Bitte:

- Beschreibe die wichtigsten Dinge in der Szene → Was passiert?
- 1-2 Saetze sind ausreichend
- Beginne den Satz NICHT mit ‚Es gibt..‘
- Beschreibe KEINE unwichtigen Details
- Beschreibe KEINE Dinge, die moeglicherweise in der Vergangenheit oder Zukunft passieren
- Beschreibe NICHT, was eine Person moeglicherweise sagt
- Gib Personen KEINE Namen
- Jeder Satz sollte mindestens 8 Woerter lang sein
- Achte darauf, Verben zu benutzen

Keine Sorge, ich werde mit darauf achten, dass Du Dich daran erinnerst. Am allerwichtigsten ist, dass Du versuchst, den Kopf in der Kinnstuetze so still wie moeglich zu halten.

Zwei Beispielbilder und die jeweilige Beispielbeschreibung wären:

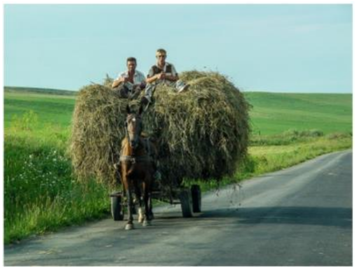

1. „Ein Pferd trägt eine große Ladung Heu und zwei Personen, die darauf sitzen.“

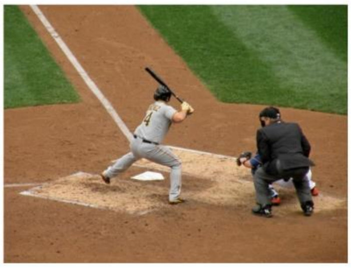

2. „Der Spieler macht sich bereit, den Ball zu schlagen, während der Schiedsrichter zuschaut.“

Translated instructions

It is important that you do not move your head in the chinrest.

In each trial, you will first see a fixation cross. Once you look at it and press the spacebar, you will see an image for 3 seconds, which you can freely view.

Afterwards, we will ask you to describe this image orally, and I will transcribe your description. There are no right or wrong answers.

Please:

- Describe the most important things in the scene → What is happening?
- 1-2 sentences are sufficient.
- Do not start the sentence with 'There is...'
- Do not describe unimportant details.
- Do not describe things that may happen in the past or future.
- Do not describe what a person might say.
- Do not give names to people.
- Each sentence should be at least 8 words long.
- Make sure to use verbs.

Don't worry; I will remind you to follow these guidelines. Most importantly, try to keep your head as still as possible in the chinrest.

Two example images and their respective example descriptions would be:

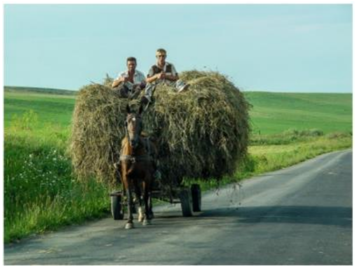

1. "A horse carries a large load of hay with two people sitting on it."

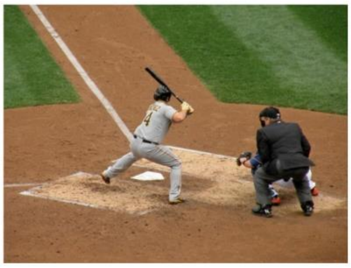

2. "The player prepares to hit the ball while the referee watches."

Supplementary Information B

Original sentences that were translated to English in Figure 2.

01: Zwei Frauen sitzen in einer Bar und eine hat eine Kamera in der Hand.

11: Zwei Frauen sitzen in einer Bar, eine von ihnen hat eine Kamera in der Hand.

09: Mehrere Personen sitzen in einem Irish-Pub, eine Person hat eine Kamera in der Hand.

19: Eine Frau sitzt auf einer Bank und hält eine Kamera. Neben ihr sitzt eine weitere Frau.

Original sentences that were translated to English in Figure 3.

26: Zwei Frauen sitzen auf einer Bank. Die Frau, die eine Kamera in der Hand hält, grinst mit ausgestreckter Zunge in die Kamera.

27: Zwei Frauen, die in einer Kneipe waren, im Hintergrund war ein Schild mit Irland zu erkennen. Die Frauen sahen amüsiert aus.

Supplementary Information C

In the main text, the Gaze Dissimilarity Matrix was calculated based on predefined object pixel masks that can be most directly associated with nouns and thus could have biased our results. For this reason, we conducted an additional control analysis using unrestricted Gaussian-smoothed fixation density maps (fixDensityGDM) for each individual. These maps were compared across participants and the correlations averaged across images for each pair of observers. We found high reliability across odd- and even-image splits for this fixDensityGDM ($r = .81$, $p < .001$). Furthermore, fixDensityGDM correlated positively with both the DDM for nouns ($r = .49$, $p < .001$) and complete descriptions ($r = .29$, $p < .001$), but not with the DDMs for verbs ($r = -.12$, $p = .01$) or adjectives ($r = -.07$, $p = .152$), replicating our original results. The same pattern was observed when weighting fixations in the density maps by duration (fixDurationWeightedDensityGDM; split-half reliability $r = .84$, $p < .001$; DDM nouns: $r = .51$, $p < .001$, complete descriptions: $r = .33$, $p < .001$, verbs: $r = -.13$, $p = .007$, adjectives: $r = -.04$, $p = .393$).

Notably, when using fixation density maps (both weighted and unweighted by fixation duration, see Figure S1 for a comparison), the correlation with DDM nouns even increased. However, their correlation with DDM verbs remained insignificant or even negative, which is in line with our main results. Here, the negative correlation was even significant, which is challenging to interpret, suggesting that greater gaze dissimilarity between participants corresponds to greater similarity in verb usage. As with the GDM based on object dwell times, we again found no significant relationship between fixation density-based GDMs and DDMs for adjectives.

We conclude that our main findings are robust to using object or pixel-based gaze similarity metrics: Inter-individual gaze similarity is aligned with the usage of nouns, but not verbs or adjectives.

Figure C1

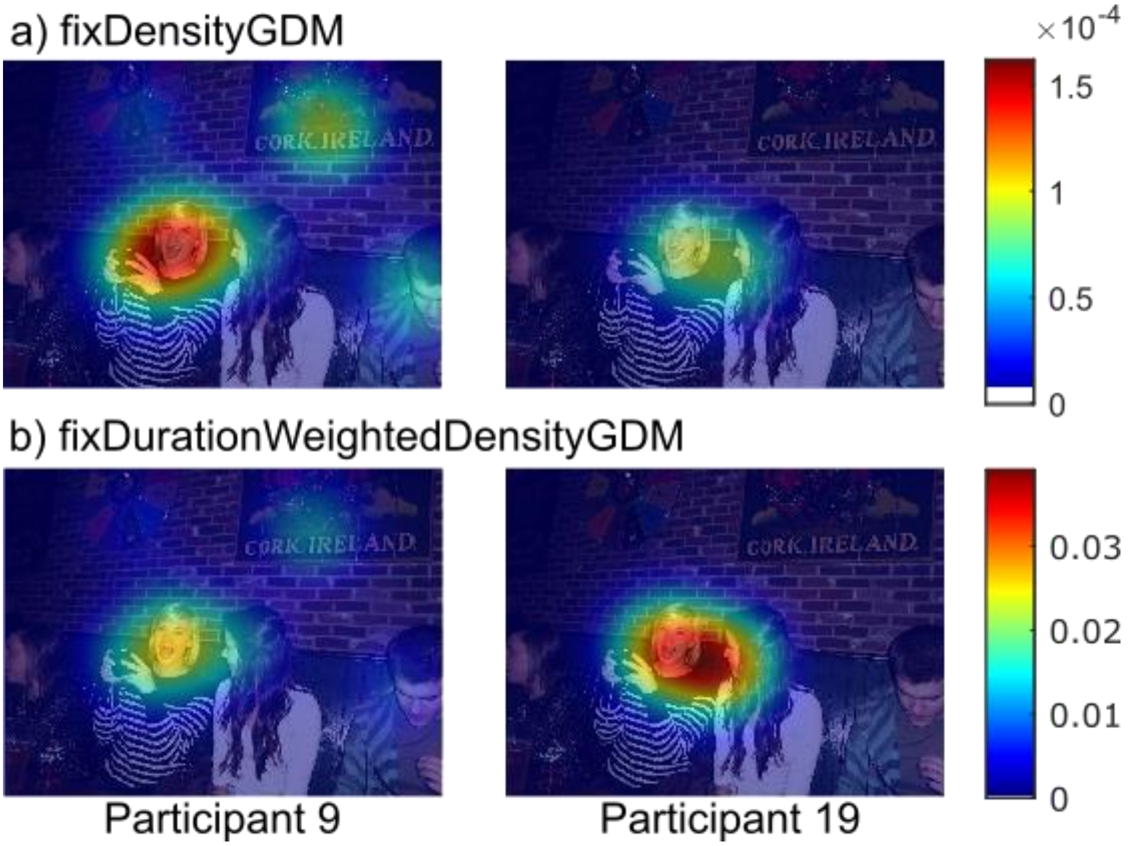

Example fixation density maps for Participants 9 and 19 (a dissimilar pair), displayed in separate columns. a) shows unweighted fixation density maps, while b) shows maps weighted by fixation duration.

Supplementary Information D

In the main text, we focused our analysis on gaze dissimilarity patterns related to the semantic dimensions of text and people and their correlation with description patterns. Previous research suggests that individual gaze tendencies toward text and faces are particularly robust in smaller stimulus sets, such as the 100 images used in this study (cf. Linka & de Haas, 2020). However, gaze tendencies toward other semantic categories, such as emotion, taste, motion, touch, and gaze, may also correlate with description patterns involving nouns, verbs, or adjectives.

Here, we present an exploratory analysis of the internal consistencies of these semantic dimensions. For dimensions with $r > .25$, we examined their correlations with the respective DDMs and reported these results in Table D1. The GDMs for taste and motion correlated

positively with DDMnoun only, whereas GDMtouched showed positive correlations not only with DDMnoun but also with DDMs for verbs and adjectives.

Table D1

Internal Consistencies of Gaze Dissimilarity Across Five Semantic Dimensions (Emotion, Taste, Motion, Touched, Gazed) and Their Correlations with Description Dissimilarity

Semantic dimension	Number of objects labeled	Internal consistency	Correlation with DDM*noun*	Correlation with DDM*verb*	Correlation with DDM*adj*
GDM*emotion*	41	*R* = .09, *p* = .060			
GDM*taste*	50	*R* = .32, *p* < .001	*R* = .41, *p* < .001	*R* = .06, *p* = .209	*R* = -.12, *p* = .010
GDM*motion*	99	*R* = .46, *p* < .001	*R* = .26, *p* < .001	*R* = -.09, *p* = .070	*R* = -.02, *p* = .675
GDM*touched*	105	*R* = .25, *p* < .001	*R* = .13, *p* = .006	*R* = .13, *p* = .006	*R* = .10, *p* = .033
GDM*gazed*	62	*R* = .14, *p* = .003			

Note. GDM = Gaze dissimilarity matrix, DDM = description dissimilarity matrix. P-values are uncorrected for multiple comparisons.

Supplementary Information E

We were able to decode which of two observers provided a given dwell-time distribution based on their descriptions for other images (and vice versa). Here, we explored whether this was also possible when limiting the gaze data to either individual differences in text or people fixations.

We were able to decode which of two observers provided a given dwell-time distribution related to text from one set of scenes to another (GDM*text*; gaze <-> gaze, decoding hit rate: 63%, p < 0.001). However, we could not decode gaze patterns related to text in one half of the dataset based on scene descriptions in the other (and vice versa; GDM*text* and DDM*noun*; gaze <-> descriptions, decoding hit rate: 51%).

For dwell-time distributions related to people, we successfully decoded participants both within and across modalities. Specifically, we achieved a gaze <-> gaze decoding hit rate of

77% (GDM*people*; $p < 0.001$) and a gaze <-> descriptions decoding hit rate of 72% (GDM*people* and DDM*noun*; $p < 0.001$).

Figure E1

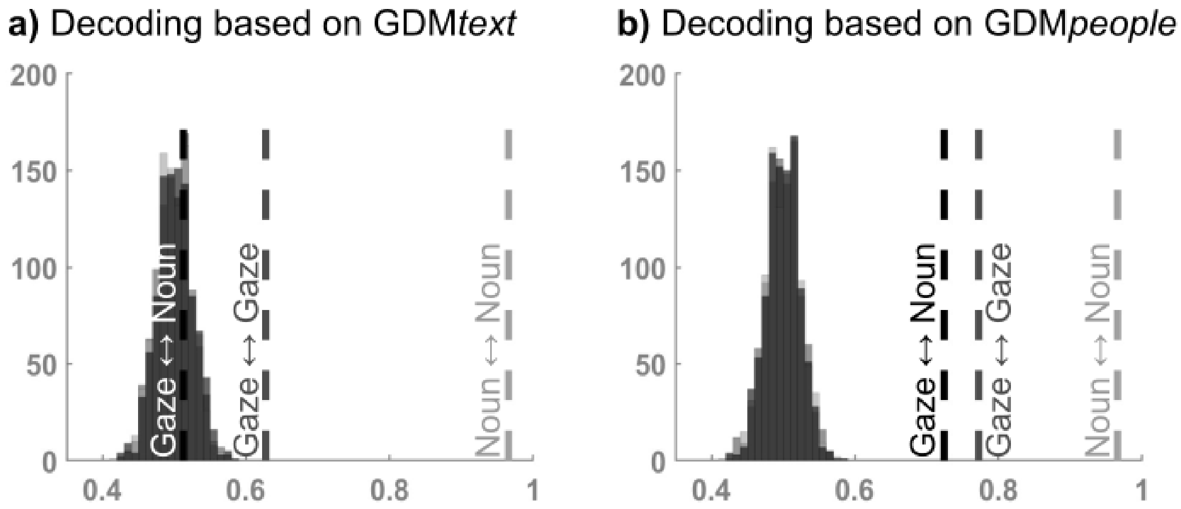

The figure shows bootstrapped null distributions and decoding hit rates based on nearest neighbour correlation between a) GDMtext and DDMnoun and b) GDMtext and DDMnoun from independent trials (odd and even splits), respectively. The vertical line of the Noun <-> noun hit rate (97%, light grey) is the same in both panels and as reported in the main text.
